# CSF1/CSF1R Axis Blockade Limits Mesothelioma and Enhances Efficiency of Anti-PDL1 Immunotherapy

**DOI:** 10.3390/cancers13112546

**Published:** 2021-05-22

**Authors:** Sophia Fotiou Magkouta, Photene Christou Vaitsi, Apostolos Georgiou Pappas, Marianthi Iliopoulou, Chrysavgi Nikolaou Kosti, Katherina Psarra, Ioannis Theodorou Kalomenidis

**Affiliations:** 1 Marianthi Simou Laboratory, 1st Department of Critical Care and Pulmonary Medicine, National and Kapodistrian University of Athens, School of Medicine, Evangelismos Hospital, 3 Ploutarchou st, 2nd Floor, 10675 Athens, Greece; photenevaitsi@gmail.com (P.C.V.); apo.pappas88@gmail.com (A.G.P.); dr.mar.iliopoulou@gmail.com (M.I.); xkdocxk@gmail.com (C.N.K.); ikalom@med.uoa.gr (I.T.K.); 2 Department of Immunology—Histocompatibility, Evangelismos Hospital, 10675 Athens, Greece; katherina@kyttarometria.gr

**Keywords:** mesothelioma, immunotherapy, CSF1R, macrophages

## Abstract

**Simple Summary:**

CSF1/CSF1R signaling mediates tumor-associated macrophages recruitment and M2 polarization. M2 TAMs are dominant immune populations infiltrating mesothelioma tumors. We evaluated the role of CSF1/CSF1R axis blockade in tumor-infiltrating immune subsets. We also examined the effect of combined anti-CSF1R and anti-PDL1 treatment in mesothelioma progression. We show that CSF1R inhibition impedes mesothelioma progression, abrogates infiltration of TAMs, facilitates an M1 anti-tumor phenotype and activates tumor dendritic and CD8+ T cells. We also show that this inhibitor was able to significantly improve the effectiveness of anti-PDL1 immunotherapy.

**Abstract:**

Colony-Stimulating Factor 1 (CSF1)/Colony-Stimulating Factor Receptor 1 (CSF1R) signaling orchestrates tumor-associated macrophage (TAM) recruitment and polarization towards a pro-tumor M2 phenotype, the dominant phenotype of TAMs infiltrating mesothelioma tumors. We hypothesized that CSF1/CSF1R inhibition would halt mesothelioma growth by targeting immunosuppressive M2 macrophages and unleashing efficient T cell responses. We also hypothesized that CSF1/CSF1R blockade would enhance the efficacy of a PDL1 inhibitor which directly activates CD8+ cells. We tested a clinically relevant CSF1R inhibitor (BLZ945) in mesothelioma treatment using syngeneic murine models. We evaluated the role of CSF1/CSF1R axis blockade in tumor-infiltrating immune subsets. We examined the effect of combined anti-CSF1R and anti-PDL1 treatment in mesothelioma progression. CSF1R inhibition impedes mesothelioma progression, abrogates infiltration of TAMs, facilitates an M1 anti-tumor phenotype and activates tumor dendritic and CD8+ T cells. CSF1R inhibition triggers a compensatory PD-1/PDL1 upregulation in tumor and immune cells. Combined CSF1R inhibitor with an anti-PDL1 agent was more effective in retarding mesothelioma growth compared to each monotherapy. In experimental mesotheliomas, CSF1R inhibition abrogates tumor progression by limiting suppressive myeloid populations and enhancing CD8+ cell activation and acts synergistically with anti-PDL1.

## 1. Introduction

Malignant pleural mesothelioma is the most common primary pleural tumor with an increasing global incidence and dismal prognosis [1]. Current treatment methods for mesothelioma are unsatisfactory [2,3]. Immunotherapy has recently attracted investigators’ interest as a potential therapeutic approach for mesothelioma [4]. Albeit early findings with PD-1/PDL1 targeting agents seem promising [5,6,7,8,9], many important issues remain unresolved, i.e., ideal drug or drug combinations, proper selection of patients most likely to respond, etc.

Tumor-Associated Macrophages (TAMs) present the dominant innate immune population of the mesothelioma inflammatory tumor infiltrate [10], and they are mainly M2-polarized [11] and highly immunosuppressive, with >75% of them expressing PDL1, consisting the main source of PDL1 in a tumor microenvironment [10]. Colony-Stimulating Factor 1 (CSF1)/Colony-Stimulating Factor Receptor (CSF1R) signaling plays important role in the recruitment of monocytes into mesothelioma tumors, their differentiation into M2-like macrophages [11,12], impaired CD8 T cell cytotoxicity [12] and chemoresistance [11,13]. In the clinic, abundant macrophage infiltration [14,15], CSF1 pleural levels [12] and PDL1 expression [16] have been linked to poor prognosis of mesothelioma patients. Taken together, the aforementioned findings support a multipotent role of the CSF1/CSFR1 axis in mesothelioma-infiltrating immune cells and provide a strong rationale for investigating whether CSF1/CSFR1 blockage would impair mesothelioma progression in vivo. We hypothesized that CSF1/CSF1R inhibition would limit mesothelioma progression by targeting immunosuppressive macrophages. We also hypothesized that CSF1/CSF1R blockade would enhance the efficacy of PDL1 inhibitor.

Our hypotheses were tested on two murine syngeneic models using a highly selective small molecule CSF1R inhibitor (CSF1Ri, BLZ945). We documented that CSF1R inhibition impedes mesothelioma progression, abrogates macrophage tumor infiltration, polarizes the remaining ones towards an M1 anti-tumor phenotype and activates tumor dendritic and T cells. We demonstrated that mesotheliomas responded to CSF1R inhibition by upregulating their PDL1 expression and that combining the CSF1Ri with a PDL1 inhibitor was more effective in retarding mesothelioma growth compared to each monotherapy.

## 2. Materials and Methods

In vitro studies: AE17 and AB1 murine mesothelioma cells [17,18] were kindly provided by Dr, YCG Lee, Perth, Western Australia and maintained as previously described [19]. Both cell lines used in the present study were periodically monitored for mycoplasma presence by PCR. Their morphology was examined on a regular basis.

BLZ945, a highly selective small-molecule inhibitor of CSF1R (hereinafter referred to as CSF1Ri), was provided by Novartis Pharmaceuticals (Basel, Switzerland). BLZ945 was freshly prepared and formulated at a concentration of 12.5 mg/mL in a vehicle that consisted of hydroxypropyl-β-cyclodextrin (HPβCD, 20% w/v) dissolved in H2O and was given at a dose of 200 mg/kg p.o (by oral gavage) once daily [20] starting from day 4 when pleural tumors are evident [21]. InVivoMAb anti-mouse PDL1 (B7-H1) (Clone: 10F.9G2) was purchased from BioxCell (West Lebanon, USA). This neutralizing antibody was administered at a dose of 200 μg i.p., every 3 days. This regimen has been reported to present a weak inhibition of AB1 tumors [22].

To investigate whether the CSF1/CSF1R axis impacts tumor cell growth, 3 × 10^3^ tumor cells were seeded at 96-well plates, and 24 h later, they were treated with CSF1 (10–20 ng/mL), BLZ945 (670 nM) or the vehicle for 24 h. To investigate whether IL34 (an alternative ligand of CSF1R) might affect tumor cell viability, we repeated experiments using the vehicle, IL34 (1 and 10 μg/mL, found to affect lymphoma tumor cell growth, [23]) and BLZ945. Cell viability was subsequently evaluated by MTS reduction (Promega, Madison, WI, USA).

CSF1 expression by mesothelioma cell lines was quantified from confluent cultures by ELISA according to manufacturers’ instructions (CUSABIO Technology LLC, Houston, TX, USA) [24].

In vivo studies

**Murine models:** C57BL/6 and Balb/c mice were purchased from BSRC Al. Fleming (Vari, Greece). All strains were housed at the Animal Model Research Unit of Evangelismos Hospital, receiving food and water ad libitum. Experiments were approved by the Veterinary Administration Bureau, Prefecture of Athens, Greece (Decision No: 7727, 30/11/2016) under compliance with the national law and the EU Directives.

AE17 or AB1 (5 × 10^5^) mesothelioma cells were intrapleurally injected to 8–10-week-old C57BL/6 or Balb/c syngeneic mice, respectively [25]. Four days upon tumor cell implantation, animals were divided into two groups, receiving either the vehicle or the CSF1R inhibitor BLZ945. In experiments testing the beneficiary effects of combinatory treatment, mesothelioma-bearing mice were split into 4 groups: control group, CSF1Ri group, anti-PDL1 group and anti-CSF1R + anti-PDL1 group. BLZ945 administration started on day 4, and anti-PDL1 commenced on day 5 after tumor cell inoculation.

Animals were euthanized 12–14 days after pleural delivery of tumor cells. Mesothelioma tumors were collected and weighed, while pleural fluid was retrieved and quantified.

**Flow cytometry:** Tumor and pleural immune cells were fixed, permeabilized and stained with anti-CD45 (30-F11), CD11b (M1/70), F4/80 (BM/8), CD206 (C068C2), Ly6C (HK1.4), Ly6G (RB6-8C5), CD11c (HL3), MHCII (M5/114.15.2), IL10 (JES5-16E3), IL12 (C15.6), CSF1R (AFS98), CD3 (145-2C11), CD4 (GK1.5), CD8 (YTS1567.7), Foxp3 (MF14), granzyme-B (QA18A28), PD-1 (29F.1A12) and PDL1(10F.9G2) (all purchased from Biolegend, San Diego, CA, USA). Inflammatory cells were selected based on their forward and side scatter profiles and their CD45 positive staining. In specific, macrophage populations were characterized as CD11b+/Ly6G−/Ly6Clow/F4/80+, M-MDSCs as CD11b+/Ly6G-/Ly6C, PMN-MDSCs as CD11b+/Ly6G+/Ly6Clow and activated DCs as CD11c+/MHCII+ cells. M2 macrophage phenotype was determined as F4/80+/CD206+. In addition, M1/M2 phenotypes were evaluated according to macrophage IL-12/IL-10 expression ratio. Total numbers of CD3+/CD4+ and CD3+/CD8+ lymphocytes were also enumerated. T-regulatory cells were determined as CD4+/Foxp3+, as well as activated CD8+ T-cells by granzyme-B expression (the gating strategy is displayed in Figure S4A–C). The flow cytometry data were acquired using BD FACSCantoII flow cytometer and analyzed by FlowJo Software (LLC, Ashland, OR, USA).

**Immunohistochemistry and immunofluorescence analyses:** Formalin-fixed, paraffin-embedded tumor tissues were stained using primary antibody rabbit anti-Proliferating Cell Nuclear Antigen (PCNA; Cell Signaling, Danvers, MA, USA) at 1:8.000 dilution for evaluation of tumor cell proliferation. Vessel density was determined upon CD31 staining of endothelial cells (1:50, polyclonal, ab28364, Abcam, Bristol, UK). Biotinylated goat anti-rabbit IgG, ABC complex kit and DAB substrate kit (Vector Laboratories) were used for detection and visualization. Tumor cell apoptosis was estimated by a TUNEL assay, as previously described [19].

For immunofluorescence analysis, tumor tissues were fixed in PFA 4% overnight at 4 °C and then transferred to 30% sucrose at 4 °C. Cryosections were stained for the presence of PDL1 (B7-H1, Biolegend, San Diego, CA, USA) and mounted using Fluoroshield Mounting Medium With DAPI (ab104139 abcam, Bristol, UK) and analyzed using Fiji software (National Institutes of Health, Bethesda, MD, USA).

**Human mesothelioma RNA-seq data analysis:** Mesothelioma gene-level raw expression RNA-seq data produced by RSEM software (Blighe, 2019) (MESO.uncv2.mRNAseq_raw_counts.txt) along with clinical information were downloaded from https://gdac.broadinstitute.org/ (accessed on 1 September 2020). Patients were divided into four groups according to their CSF1R and CD8 tumoral mRNA levels. They were first divided according to their CD8 levels: Those whose CD8 RNA-Seq by Expectation-Maximization (RSEM) values were lower to median were characterized as ‘low,’ and those with RSEM higher to median were considered to be ‘high.’ These patients were subsequently subdivided into high or low CSF1R if their CSF1R RSEM levels were above or below the median CSF1R value, respectively. Kaplan-Meier survival analysis was performed, and the survival between cohorts was compared using log-rank tests using GraphPad Prism software (version 5.0, GraphPad Software, Inc, San Diego, CA, USA).

**Statistics:** All values are presented as mean ± standard error of the mean (SEM). Differences between groups were evaluated using the 2-tailed Student’s *t*-test, or one-way analysis of variance (ANOVA) with Bonferroni post hoc test for multiple comparisons, as appropriate. *p* values < 0.05 were considered significant. Statistical analysis was performed using the Statistical Package for the Social Sciences v.13.0.0 (IMB, Armonk, NY, USA). Interventionary studies involving animals or humans, and other studies that require ethical approval, must list the authority that provided approval and the corresponding ethical approval code.

## 3. Results

### 3.1. CSF1R Blockade Impedes Mesothelioma Tumor Progression

**Figure 1:**
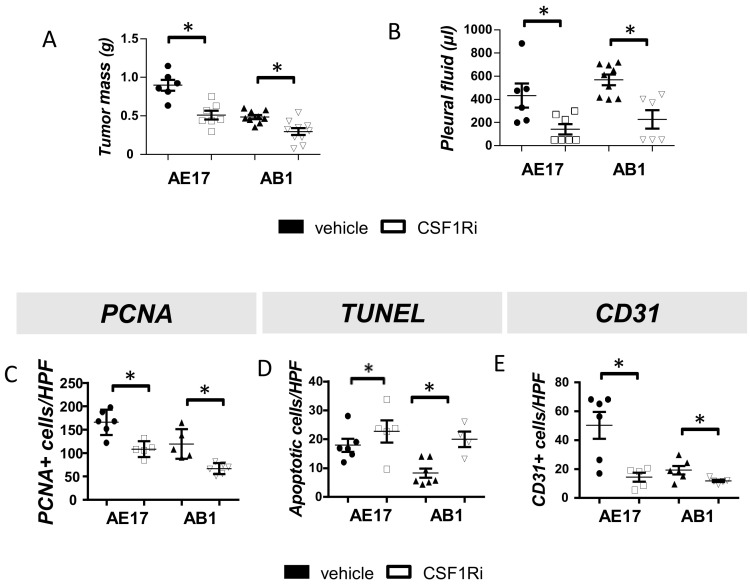
CSF1R inhibition attenuates murine mesothelioma progression by abrogating tumor cell proliferation and tumor angiogenesis and enhancing tumor cell apoptosis in vivo. AE17 and AB1 cells were intrapleurally injected into syngeneic C57Bl/6 and Balb/c mice, respectively. Mice were given BLZ945 inhibitor (200 mg/kg b.w., p.o.) or vehicle once daily. Fourteen days later, the mice were sacrificed, and the tumors were excised and weighed (**A**), and the pleural fluid was retrieved and quantified (**B**). Data presented as mean ± SEM, *n* = 6–10 from three independent experiments, * *p* < 0.05 compared to vehicle by 2-tailed students’ *t*-test. (**C**) Proliferating tumor cells were visualized upon staining for PCNA. (**D**) Tumor cell apoptosis in tumor tissues was evaluated by a TUNEL assay. (**E**) Tumor angiogenesis was evaluated in tissue sections from the vehicle- or BLZ945-treated animals upon CD31 staining. Data are presented as mean ± SEM, *n* = 5–6, * *p* < 0.05 compared to vehicle by 2-tailed students’ *t*-test. HPF: High Power Field.

To investigate whether targeting of CSF1R signaling could halt mesothelioma progression, we administered CSF1R inhibitor (or vehicle) to mesothelioma-bearing mice. CSF1Ri significantly reduced tumor burden and limited pleural fluid accumulation (Figure 1A,B). CSF1R inhibition conferred a significantly reduced tumor cell proliferation (Figure 1C and Figure S1A) and increased tumor cell apoptosis rates (Figure 1D and Figure S1B). Finally, tumors from CSF1Ri administered mice exhibited lower vessel density compared to the vehicle-treated ones (Figure 1E and Figure S1C).

**Figure 2:**
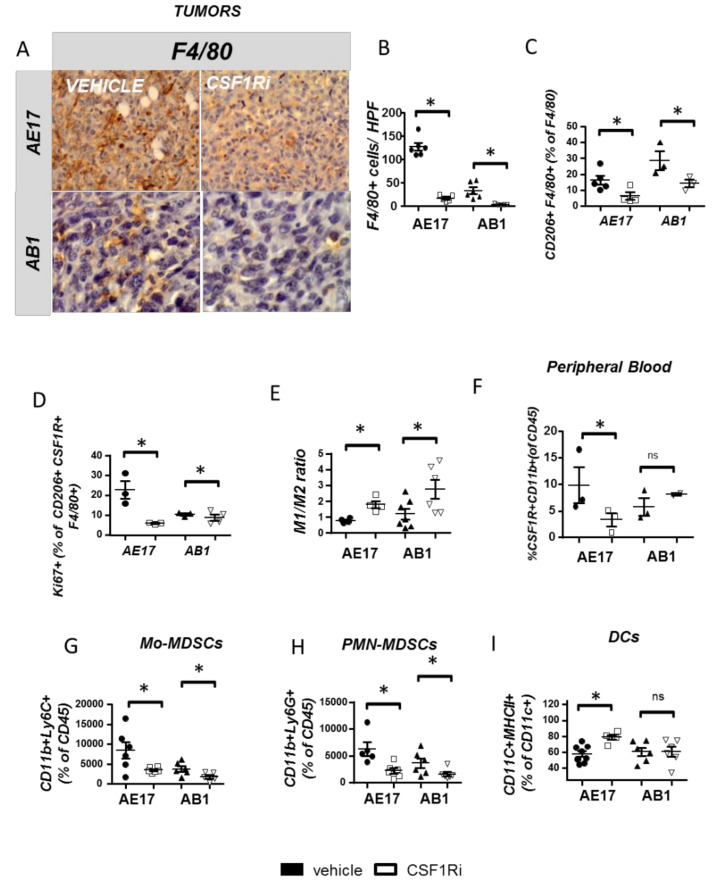
CSF1R inhibition reduces myeloid cell infiltration of mesothelioma tumors, reprograms TAMs towards an anti-tumor profile and boosts DC activation. Tumor tissue sections from vehicle- or CSF1Ri-treated animals were stained for F4/80, and macrophages were counted. (**A**) Representative pictures and (**B**) results of immunohistochemical staining analysis. Data presented as mean ± SEM, *n* = 5–6 from two independent experiments, * *p* < 0.05 compared to the vehicle by 2-tailed students’ *t*-test. HPF: High Power Field. (**C**–**I**) Tumors of vehicle- and CSF1Ri-treated animals were analyzed for major CSF1R-expressing myeloid populations using flow cytometry. (**C**) CD206+ M2 populations were quantified in tumors of vehicle- and CSF1Ri-treated mice. (**D**) M2 TAM proliferation was quantified. (**E**) TAM IL12/IL10 expression ratio (indicative of M1/M2 polarization) was determined. (**F**) Circulating CSF1R+n monocytes (CD11b+/CD45+) were quantified in the peripheral blood. (**G**,**H**) Tumor infiltration of myeloid-derived suppressor cells. (**I**) Activation (MHCII+) of tumor dendritic cells (CD11c+) was determined. Data are presented as mean ± SEM, *n* = 5–8 from two independent experiments, * *p* < 0.05 compared to the vehicle by 2-tailed students’ *t*-test. ns = not significant.

Although both mesothelioma cell lines were found to secrete CSF1 (Figure S2A) and express CSF1R (by a small percentage of the cells, Figure S2B), neither CSF1 nor CSF1Ri affected their survival in vitro (Figure S2C,D). Similarly, alternative activation of CSF1R by IL34 did not affect tumor cell viability (Figure 2E,F). The above data argue against an autocrine or paracrine impact of the CSF1/CSF1R axis on tumor cells themselves. We, therefore, assumed that the mesothelioma-limiting properties of the inhibitor should be attributed to its effects on tumor microenvironment.

### 3.2. CSF1R Inhibition Critically Reduces Tumor Promoting Myeloid Cell Populations and Reprograms TAMs towards an Anti-Tumor Phenotype

Having observed that the mesothelioma-limiting effects of CSF1R inhibitor might more likely occur as a result of its effect on tumor milieu, we focused on myeloid populations that are prevalent in mesothelioma tumors and affected by CSF1R signaling. First, we observed a profound reduction of tumor macrophages at CSF1Ri administered animals (Figure 2A,B). Intriguingly, untreated AE17 tumors presented higher macrophage infiltration then the AB1 ones (Figure 2A,B). Tumors’ peripheral and central areas were similarly affected. The inhibitor reduced M2 TAMs (Figure 2C) by inhibiting their expansion (Figure 2D) and/or enhancing their IL-12 associated M1 phenotype (Figure 2E). Recapitulating the in vivo observations, untreated M2 macrophages stimulated mesothelioma cell growth in co-culture while CSF1Ri-treated M2 macrophages and M1 macrophages reduced it (Figure S3). CSF1Ri reduced circulating CD11b+/CSF1R+ monocytes (Figure 2F) in AE17 (not in AB1) model. Apart from the effects on mature F4/80 macrophages, CSF1Ri significantly limited the number of tumor-promoting Myeloid-Derived Suppressor Cells (MDSCs) (known to express CSF1R, 22): both Mo-MDSCs (CD11b+/Ly6C+, Figure 2G) and PMN-MDSCs (CD11b+/Ly6G+, Figure 2H) subpopulations were significantly reduced upon CSF1Ri administration. Finally, CSF1Ri enhanced MHCII expression by tumoral dendritic cells (Figure 2I), which express CSF1R, too [23]. Pleural fluid macrophages, Mo-MDSCs and PMN-MDSCs, were also significantly reduced (Figure S5A,C,D, respectively). Consistent with our findings in the tumor, pleural TAMs were also polarized towards an M1 phenotype (Figure S5B).

### 3.3. CSF1R Inhibition Stimulates CD8+ Cell Activation

**Figure 3:**
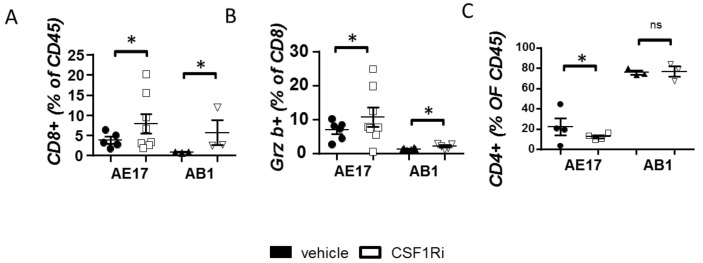
CSF1R inhibition enhances CD8 lymphocyte activation and recruitment and reduces T regulatory populations. Tumors of vehicle- and CSF1Ri-treated animals were analyzed for central T populations using flow cytometry. The total (**A**) and (**B**) activated (GranzymeB+) tumor CD8+ lymphocyte numbers were determined. (**C**) Suppressive (Foxp3+) CD4+ lymphocytes were also quantified. Data are presented as mean ± SEM, *n* = 5–8 from two independent experiments, * *p* < 0.05 compared to the vehicle by 2-tailed students’ *t*-test.

Having seen that CSF1R blockade successfully limited immune-suppressive myeloid populations, we subsequently examined whether it affected tumor lymphocytes. CD8 cell tumor infiltration (Figure 3A) and activation (Figure 3B) were increased in treated animals (Figure 3B,C). CSF1Ri-treated mice bearing AE17 but not AB1 tumors presented reduced Treg populations (Figure 3C). The aforementioned effects of CSF1Ri in the tumor lymphoid populations were also found in pleural lymphocytes (Figure S6A–E).

### 3.4. CSF1R Inhibition Triggers a Compensatory Upregulation of PDL1 in Mesothelioma and Myeloid Cells and of PD-1 on T Cells

**Figure 4:**
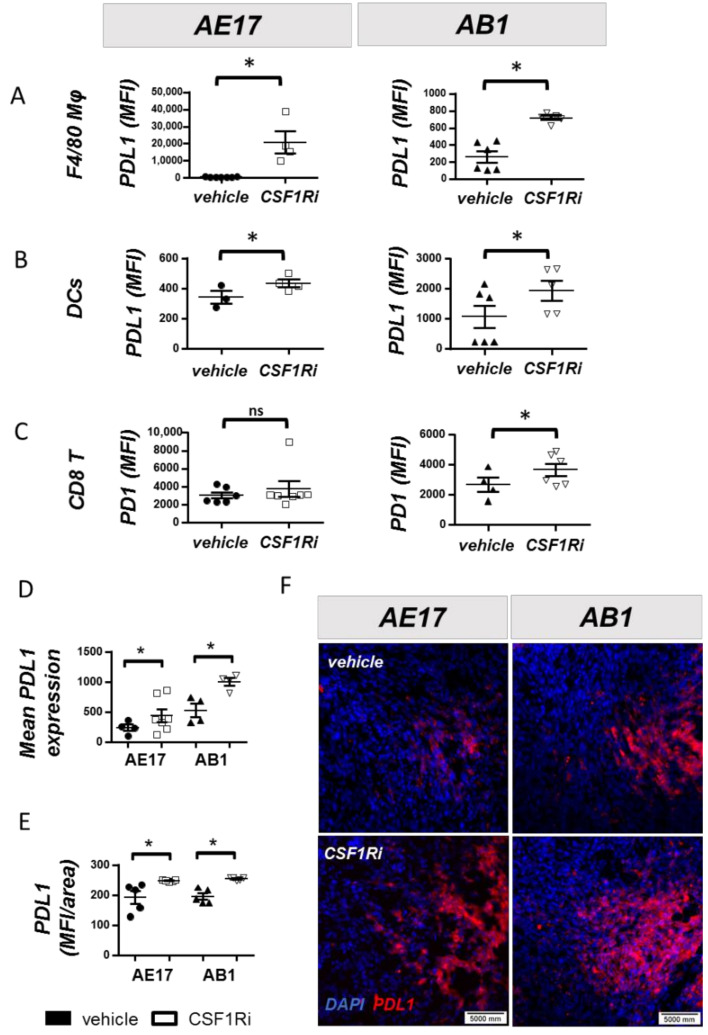
CSF1R blockade triggers a compensatory upregulation of the PD-1/PDL1 immunosuppressive axis. (**A**) TAMs, (**B**) DCs and (**C**) CD8+ lymphocytes of tumors from vehicle- or CSF1Ri-treated mice were analyzed for the expression of PDL1 or PD-1. Data are presented as mean ± SEM, *n* = 5–8 from two independent experiments, * *p* < 0.05 compared to the vehicle. (**D**) PDL1 expression by tumor (CD45-negative) cells was evaluated using flow cytometry of tumor lysates. (**E**) The overall (neoplastic and immune cell) PDL1 positivity was evaluated upon immunofluorescent staining and quantified using Fiji software. Data are presented as mean ± SEM, *n* = 5–8 from two independent experiments, * *p* < 0.05 compared to the vehicle by 2-tailed students’ *t*-test.

CSF1Ri upregulated the expression of PDL1 by macrophages (Figure 4A) and DCs (Figure 4B) and the expression of PD-1 by tumor CD8+ lymphocytes in the case of AB1 tumors (Figure 4C). Pleural fluid macrophages of treated mice also presented higher levels of PDL1 (Figure S7A). PDL1 was also increased in pleural DCs of CSF1Ri-treated animals bearing AE17 tumors (Figure S7B). PD-1 expression on CD8 T cells was not altered (Figure S7C).” As shown in Figure 4D, following the CSF1R blockade, PDL1 was significantly upregulated on mesothelioma tumor cells. The overall (neoplastic and immune cell) PDL1 positivity was visualized in tumor sections (Figure 4E,F).

Taken together, the aforementioned results imply that the observed anti-tumor responses imposed by CSF1R inhibition induced a compensatory upregulation of PD-1/PDL1 immune escape signals, which may partially limit the anti-tumor effect of the inhibitor. These data provide a rational basis for a combinatory administration of CSF1Ri and immune checkpoint inhibitors.

### 3.5. CSF1R Inhibition Amplifies the Efficacy of Immune Checkpoint Therapy in Mesotheliomas In Vivo

**Figure 5:**
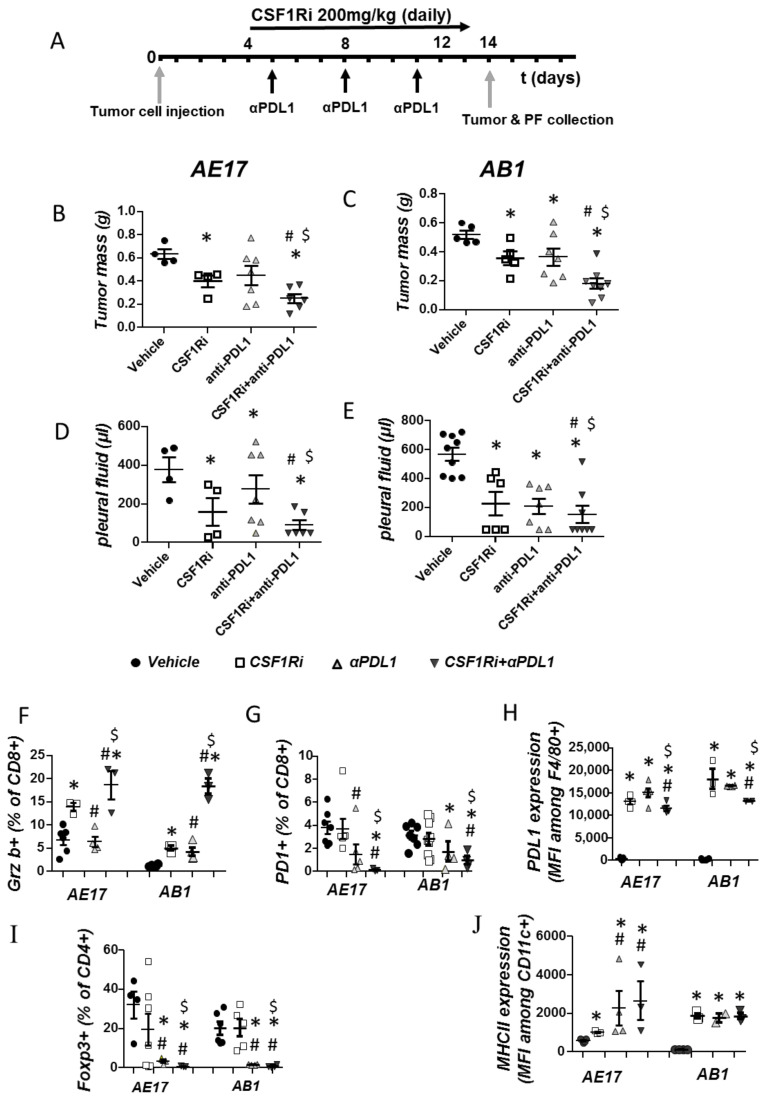
Combined CSF1R and PDL1 blockade abrogates mesothelioma progression by unleashing CD8 activation and by reducing suppressive Tregs. (**A**) AE17 and AB1 cells were intrapleurally injected into syngeneic C57Bl/6 and Balb/c mice, respectively. Mice were administered with anti-PDL1 (200 μg/dose i.p) every 3 days, CSF1Ri (200 mg/kg b.w, p.o. once daily), anti-PDL1+CSF1Ri or vehicle once daily. Fourteen days later, the mice were sacrificed, and the mesothelioma tumors were excised and weighed (**B**,**C**), and pleural fluid was retrieved and quantified (**D**,**E**). Data are presented as mean ± SEM, *n* = 5–8 from two independent experiments, * *p* < 0.05 compared to vehicle, ^#^ *p* < 0.05 compared to CSF1Ri, ^$^ *p* < 0.05 compared to anti-PDL1 group by One-way ANOVA test. (**F**,**G**) Tumors were analyzed for activated (Granzyme B+) or “suppressed” (PD-1+) CD8+ lymphocytes. (**H**) The expression of suppressive PDL1 by TAMs was determined. (**I**) Tumor infiltration of suppressive T regulatory cells was evaluated. (**J**) Activation of DCs was evaluated by quantifying their MHCII expression. Data are presented as mean ± SEM, *n* = 5–8 from two independent experiments, * *p* < 0.05 compared to the vehicle, ^#^ *p* < 0.05 compared to CSF1Ri, and ^$^ *p* < 0.05 compared to anti-PDL1 group by One-way ANOVA test.

We next investigated whether combined targeting of CSF1R and PDL1 (administration scheme shown in Figure 5A) could act in a complementary manner against mesothelioma. Dual therapy was more potent than that of monotherapies (Figure 5B–E). As for tumor immune populations, the activation of cytotoxic T cells was notably enhanced (Figure 5F). This could be functionally related to the combined treatment-induced reduction of PDL1 by macrophages (Figure 5H) compared to monotherapies.

Most importantly, significant restriction of Tregs (Figure 5I) could also contribute to increased CD8 T cell activation. DC activation was significantly enhanced in the AE17 model (Figure 5J). Finally, combined treatment significantly reduced Treg populations in the pleural fluid but did not enhance DC and CD8 activation compared to CSF1Ri (Figure S8A–C). PD-1, as well as PDL1 expression, was significantly reduced in CD8 and macrophages, respectively, compared to the CSF1Ri group in the case of the AE17 model (Figure S8D,E).

### 3.6. Clinical Significance of the CSFR1 Expression and the Interplay between CSFR1 Expressing Macrophages and CD8+ Cells in Mesotheliomas

**Figure 6:**
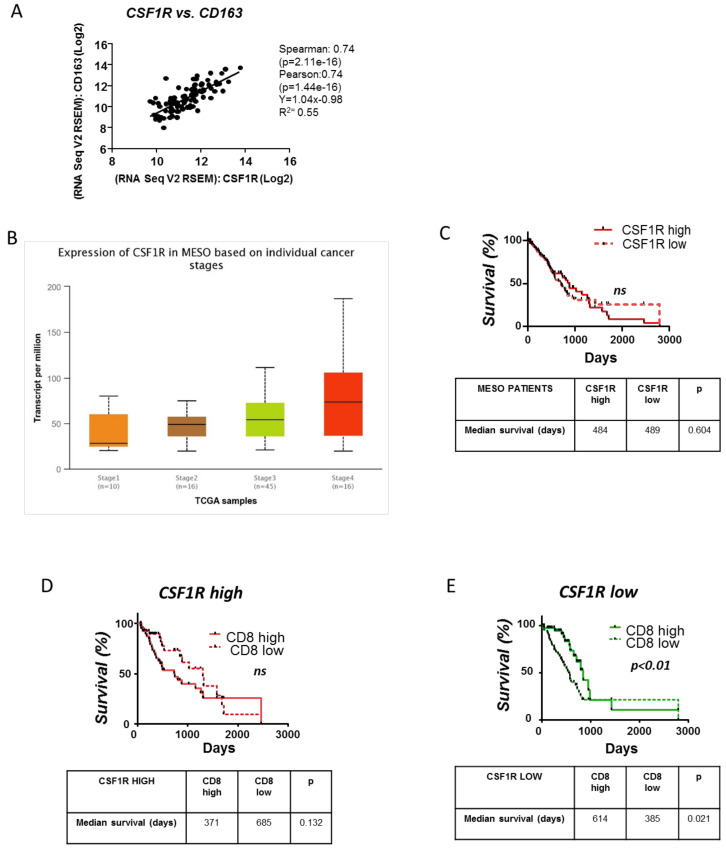
Clinical significance of the CSFR1 expression and the interplay between CSFR1 expressing macrophages and CD8+ cells in mesotheliomas. RSEM data of RNAseq analysis of 88 mesothelioma tumor samples were retrieved by the TCGA biobank. (**A**) CSF1R RSEM values correlated with CD163 (M2 marker) expression implying that CSF1R is mainly expressed by M2-like macrophages in the tumor milieu. Analysis was performed using cBioportal. (**B**) Ualcan path was used to present and analyze CSF1R RSEM values according to mesothelioma patients’ stage of the disease. (**C**) Kaplan–Meier plot linking CSF1R gene expression with mesothelioma patients’ survival. (**D**,**E**) Mesothelioma patients were divided into two groups according to CSF1R RSEM levels (high CSF1R versus low CSF1R group). Kaplan–Meier plots linking CD8 gene expression with patients’ survival (for the “high” and “low CSF1R” groups, separately) were created. Median RSEM values of CSF1R and CD8 genes were used as the cutoff to characterize “high” or “low” expression. In all cases, survival between cohorts was compared using log-rank tests using GraphPad Prism software (version 5.0) *n* = 88.

In order to obtain insights on the clinical impact of the CSF1R/CSF1 axis in mesothelioma, we investigated potential links between tumor CSF1R expression, tumor macrophages and clinical outcomes using the TCGA RNAseq data of mesothelioma patients. M2-like tumor-promoting macrophages are the major contributors of CSF1R signaling in the tumor microenvironment, as indicated by the significant correlation of the CSF1R gene with CD163 (M2 human macrophage marker) (Figure 6A).

Even though the CSF1R gene is upregulated in advanced stages of mesothelioma (Figure 6B), CSF1R expression was not associated with survival (Figure 6C). Interestingly, in patients with low CSF1R-expressing tumors, increased CD8 infiltration was linked with better prognosis (Figure 6E), while in those with high CSF1R-expressing tumors, CD8 infiltration had no impact on their survival (Figure 6D). In fact, the median survival of CD8 high patients tended to be shorter than CD8 low patients among those with CSF1R high tumors (Figure 6D), implying that in the presence of CSF1R macrophages, CD8 infiltration might be disadvantageous for the patient because they are rather suppressed, favoring tumor evasion. Altogether, albeit these observations do not permit definite conclusions, they strongly suggest that the abundance of CSF1R-expressing macrophages abolishes the beneficial effects of tumor-infiltrating CD8 T cells in mesothelioma patients’ survival.

## 4. Discussion

This study aimed to define the impact of CSF1/CSF1R axis blockade in experimental mesothelioma progression and its potential to enhance the efficacy of immune checkpoint therapy. Our main findings are: (1) Pharmacological targeting of CSF1/CSF1R axis: (a) attenuated tumor cell proliferation and tumor-associated angiogenesis and promoted tumor cell apoptosis to limit mesothelioma progression; (b) reduced tumor immunosuppressive myeloid cells including TAMs and MDSCs, hindered CSFR1+ M2 proliferation and polarized TAMs towards M1 phenotype; (c) increased tumor infiltration by CD8 lymphocytes, CD8 lymphocyte and DC activation, reduced Tregs but stimulated the expression of PD-1/PDL1 axis components by tumor and immune cells. (2) Combined anti-PDL1 and CSF1Ri treatment provoked CD8 activation and impaired mesothelioma progression more effectively than monotherapies. (3) In human mesotheliomas: (a) tumor CSF1R expression mainly occurs in TAMs, and it is linked with advanced stages of the disease. (b) Abundance of infiltrating CD8 lymphocytes is associated with better prognosis only in low CSF1R-expressing tumors.

Immunotherapy is actively investigated for its possible effects on mesothelioma, and researchers have so far focused on methods that directly stimulate the CD8-dependent adaptive immunity to fight cancer cells [7,8,9,26,27,28]. We here provide experimental proof-of-concept that targeting immunosuppressive innate immune cells, the most prominent immune suppressors in mesothelioma milieu [10], halts experimental mesothelioma progression. We demonstrated that targeting the CSF1/CSF1R axis, which is implicated in macrophage differentiation and polarization towards an angiogenic and pro-tumor, M2 phenotype [29,30], using a CSF1R inhibitor (currently tested against advanced solid tumors, NCT02829723), can retard mouse mesothelioma growth in vivo without affecting tumor cell proliferation in vitro and hamper tumor angiogenesis though endothelial cells do not express its target receptor [30]. Therefore, anti-mesothelioma effects of the treatment should most likely be attributed to its impact on the innate immune cells, suggestive of reversal of the immunosuppressive skewing of tumor innate immune environment: reduced TAM and MDSC tumor infiltration and CSF1R-expressing TAM proliferation, M1 polarization of the remaining TAMs and DC activation. These effects, together with the reduction of Treg populations, may, in turn, provoke the observed enhancement of the intratumoral accumulation and activation of the CD8 T-cells, the main cancer-killing lymphocyte subset. In fact, CSF1R+ macrophages are known to secrete high amounts of TGF-beta ex vivo [31], abolishing effective CD8 T cell responses [30] and maintaining Treg populations [32].

Besides its immune-activating effects, CSF1Ri induced an upregulation of the PD-1/PDL1 axis in mesothelioma: tumor cells, TAMs and tumor DCs of the treated animals exhibited increased expression of PDL1 while tumor CD8+ lymphocytes had increased expression of PD-1. This compensatory response has been also reported in pancreatic and hepatocellular tumors [24,31]. This might present compensation to the immune-stimulating properties of the CSF1Ri and may drive resistance to the treatment, similar to that observed with CD8+ cell-activating therapies for which apoptotic Treg and recruitment of MDSCs antagonize the antitumor effect of anti-PD-1/PDL1 [33,34,35]. CSF1R inhibition has been previously shown to provoke a compensatory increase in Tregs [30] and MDSCs [36]. However, in our hands, Tregs were unaffected, and MDSCs were reduced by CSF1Ri treatment. Despite PD-1/PDL1 upregulation was found to antagonize the anti-tumor effects of the CSF1Ri, it may, at the same time, uncover a potential additional anti-tumor target since PDL1 expression is a classic marker of sensitivity in anti-PDL1 drugs [37]. Not surprisingly then, dual inhibition of CSF1R and PDL1 more effectively abrogated mesothelioma progression by triggering more profound CD8+ cell and DC activation compared to monotherapies. Our findings suggest that the combination of CSF1R inhibitor with a PDL1 blocking agent carries strong potentials as an anti-mesothelioma regimen and pave the way for clinical trials to explore this possibility. The link between CSF1R expression, CD8+ cells and survival of mesothelioma patients further encourages the clinical testing of the combination.

## 5. Conclusions

In conclusion, CSF1R blockage impairs mouse mesothelioma growth by limiting suppressive (M2 TAM, MDSC and Treg) and promoting antitumor (DC and CD8+ lymphocyte) immune cell accumulation/activation. Treatment with CSF1R inhibitor resulted in a compensatory upregulation of PD-1/PDL1 pathway in tumor tissue, and its combination with anti-PDL1 was more effective than monotherapies in preventing mesothelioma growth. Since both CSFR1 and PDL1 inhibitors are under clinical investigation, our findings call for clinical testing of the therapeutical concept investigated in the present study.

## Data Availability

The datasets used and/or analyzed during the current study are available from the corresponding author on reasonable request.

## Supplementary Materials

The following are available online at https://www.mdpi.com/article/10.3390/cancers13112546/s1, Figure S1: CSF1R inhibition abrogates tumor cell proliferation and tumor angiogenesis and enhances tumor cell apoptosis in vivo, Figure S2: AE17 and AB1 mesothelioma cells secrete CSF1 in vitro and express the CSF1R inhibitor in vivo. AE17 and AB1 growth is not affected by CSF1R activation, Figure S3: CSF1R reprograms M2 macrophages towards an M1 like phenotype in vitro, Figure S4: Flow cytometry gating strategy, Figure S5: CSF1R inhibition reduces CSF1R+ macrophage numbers in mesothelioma associated pleural effusion and favors M1 TAMs polarization, Figure S6: CSF1R inhibition enhances DC activation, favors CD8 lymphocyte recruitment and activation and reduces Treg in mesothelioma associated pleural effusion, Figure S7: CSF1R blockade up-regulates PDL1 expression by pleural fluid Macrophages and DCs, Figure S8: Combined CSF1R and PDL1 blockade significantly enriches pleural fluid with activated CD8 and DC populations and reduces accumulation of suppressive ones .
