# Supplementary material for: CSF1/CSF1R Axis Blockade Limits Mesothelioma and Enhances Efficiency of Anti-PDL1 Immunotherapy
Source: Cancers (Basel). 2021 May 22;13(11):2546. doi: 10.3390/cancers13112546 (PMC8196870; doi:10.3390/cancers13112546)
Supplement: Supplementary file 1 [file cancers-13-02546-s001.zip › cancers-1197039-supplementary.pdf]

cancers

MDPI

Supplementary Files

CSF1/CSF1R Axis Blockade Limits Mesothelioma and Enhances Efficiency of Anti-PDL1 Immunotherapy

Sophia Fotiou Magkouta, Photene Christou Vaitsi, Apostolos Georgiou Pappas, Marianthi Iliopoulou, Chrysavgi Nikolaou Kosti, Katherina Psarra and Ioannis Theodorou Kalomenidis

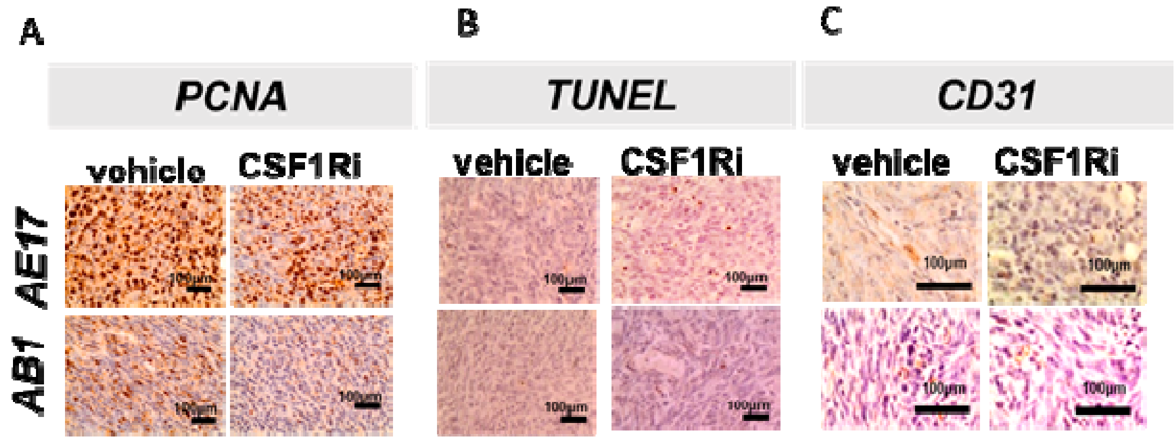

Figure S1. CSF1R inhibition abrogates tumor cell proliferation and tumor angiogenesis and enhances tumor cell apoptosis in vivo**.** AE17 and AB1 cells were intrapleurally injected into syngeneic C57Bl/6 and Balb/c mice, respectively. Mice were given BLZ945 inhibitor (200 mg/kg b.w., p.o.) or vehicle once daily. Fourteen days later mice were sacrificed and tumors were excised, weighed and stained for PCNA (**A**), CD31(**C**) or subjected to TUNEL (**B**) for apoptosis evaluation. Representative pictures of immunohistochemical analyses.

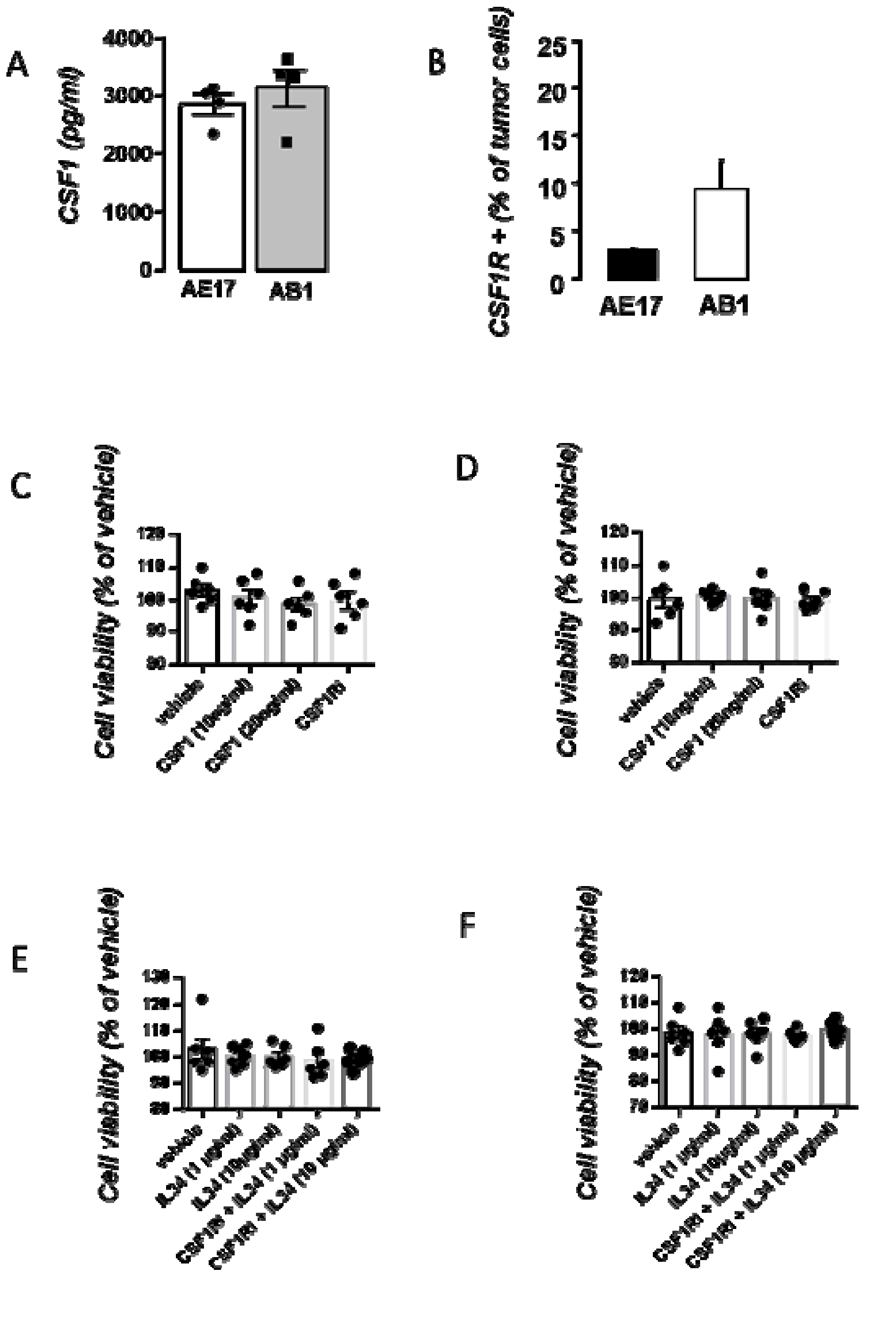

Figure S2. AE17 and AB1 mesothelioma cells secrete CSF1 in vitro and express the CSF1R inhibitor in vivo. AE17 and AB1 growth is not affected by CSF1R activation. (**A**) CSF1 expression by mesothelioma cell lines was quantified from confluent cell cultures by ELISA and (**B**) CSF1R expression was evaluated in Cytokeratin+ tumor cells isolated from AE17 and AB1 tumors upon collagenase II treatment by flow cytometry. Data are presented as mean ± SEM, *n* = 3–4. (**C**,**E**) AE17 or (**D**,**F**) AB1 cells were seeded at 3 × 10³ cells/well and treated with CSF1Ri (670 nM, a concentration that does not affect macrophage viability) and treated with CSF1 (10–20 ng/mL), BLZ945 (670 nM) or vehicle for 24 h (**C**,**D**) or vehicle, IL34 (1 and 10 μg/mL) and BLZ945 + IL34 (**E**,**F**). 24 h later tumor cell viability was measured by MTS assay. Data are presented as means ± SEM, *n* = 6, **p* < 0.05 of vehicle.

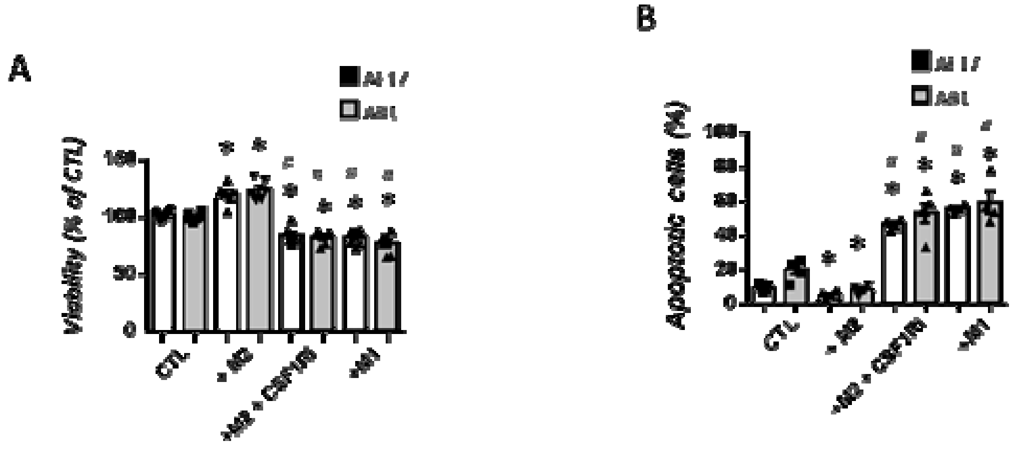

Figure S3. CSF1R reprograms M2 macrophages towards an M1 like phenotype in vitro**.** (**A**) Macrophages were obtained from bone marrow cells using treatment with CSF1 for 7 days and they were subsequently polarized towards M1 or M2 phenotype by incubation with LPS (100 ng/mL) or IL-10 (20 ng/mL) respectively for 48 h. M1 or M2 macrophages were co-cultured with equal numbers of AE17 or AB1 mesothelioma cells with or CSF1Ri (670 nM, a concentration that does not affect macrophage viability) in serum free conditions and 48h later tumor cell viability was measured by MTS assay (**B**) and tumor cell apoptosis was evaluated by Annexin V/PI staining. Data are presented as means ± SEM, n = 5–6, * $p < 0.05$ of CTL, # $p < 0.05$ of M2.

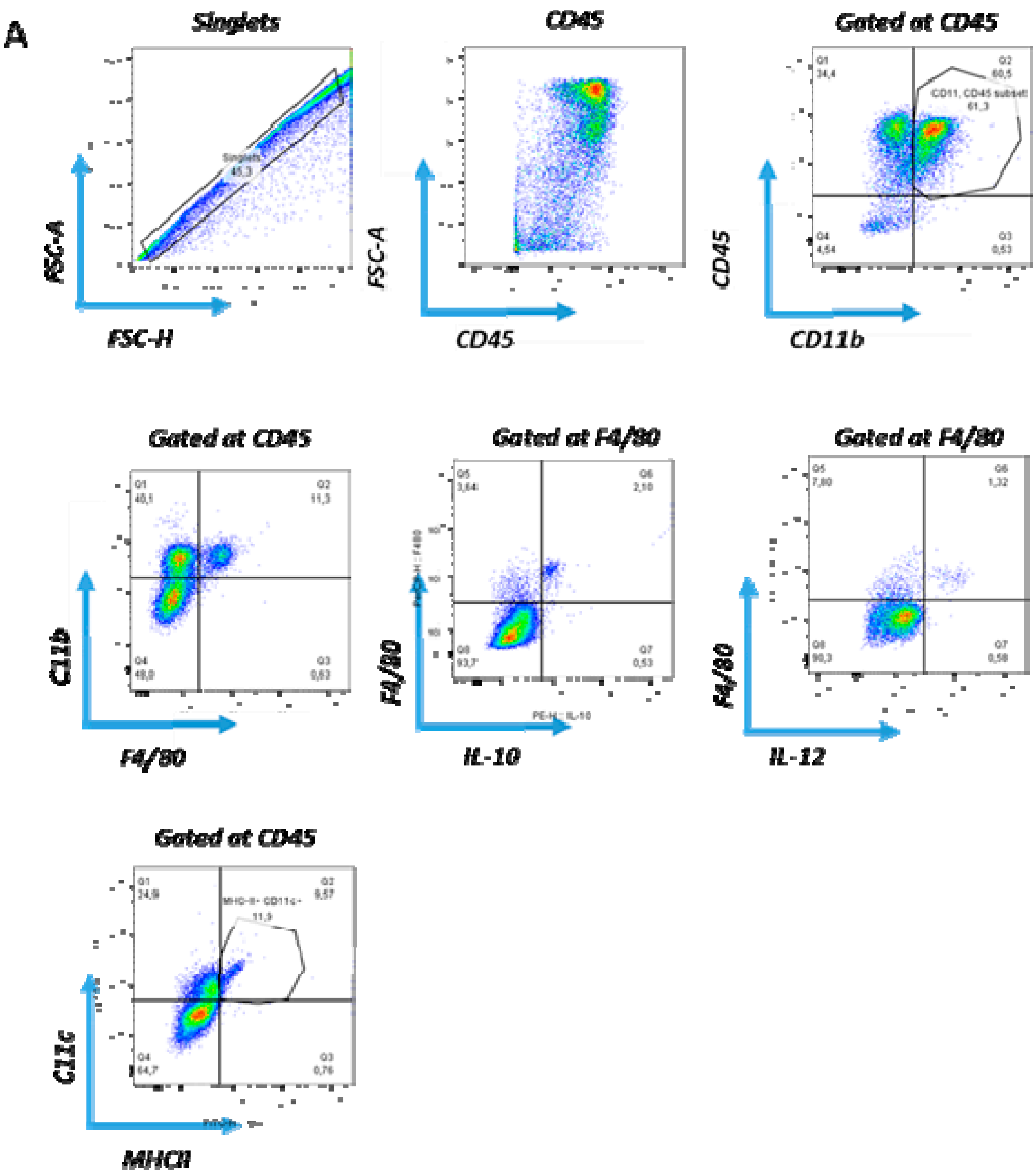
A
Singlets
FSC-A
FSC-H
CD45
FSC-A
CD45
Gated at CD45
CD45
CD11b
Gated at CD45
C11b
F4/80
Gated at F4/80
F4/80
IL-10
Gated at F4/80
F4/80
IL-12
Gated at CD45
C11c
MHCII

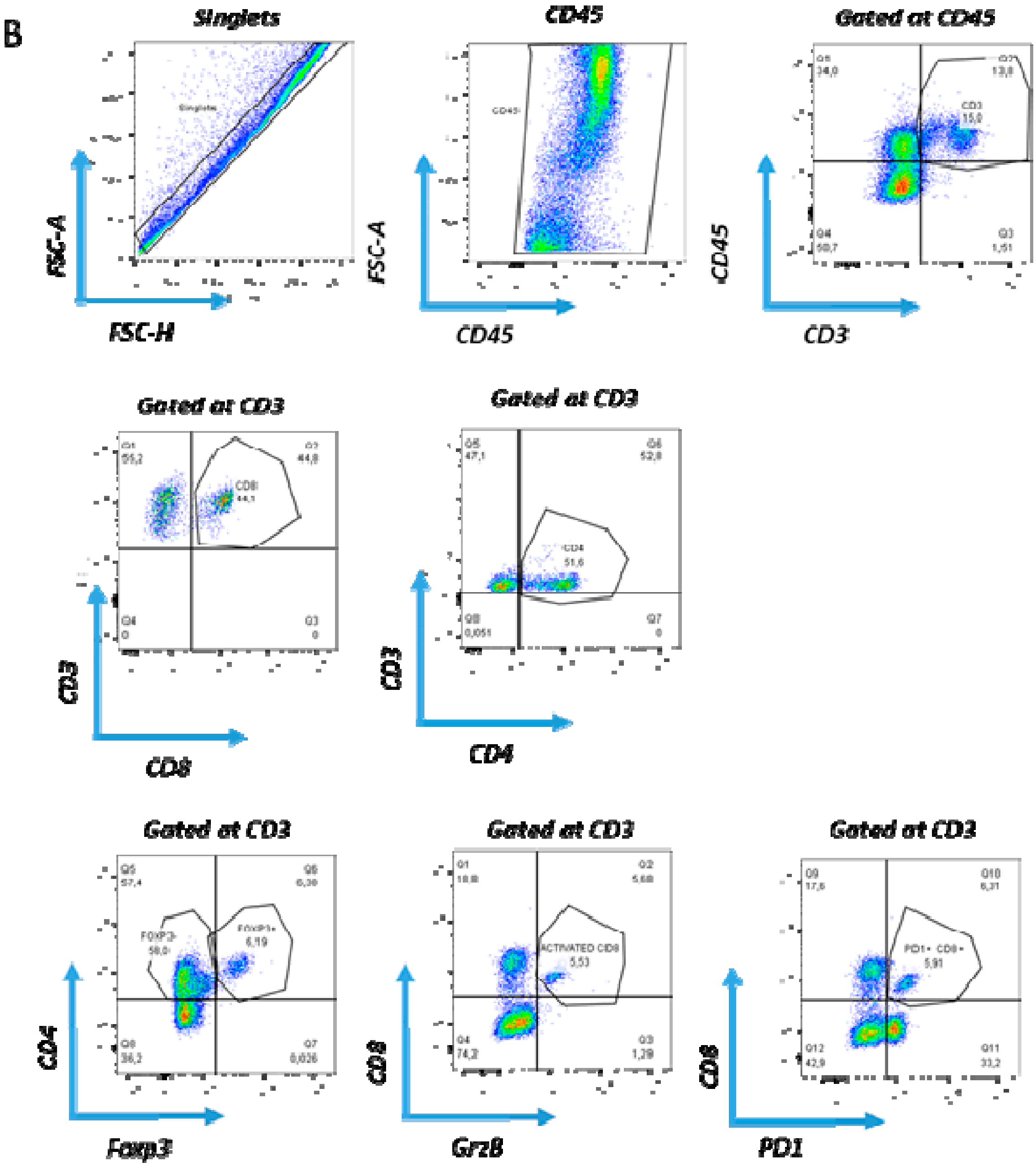
B
Singlets
FSC-A
FSC-H
CD45
FSC-A
CD45
Gated at CD45
CD45
CD3
Gated at CD3
CD3
CD8
Gated at CD3
CD3
CD4
Gated at CD3
CD4
Foxp3
Gated at CD3
CD8
GrzB
Gated at CD3
CD8
PD1

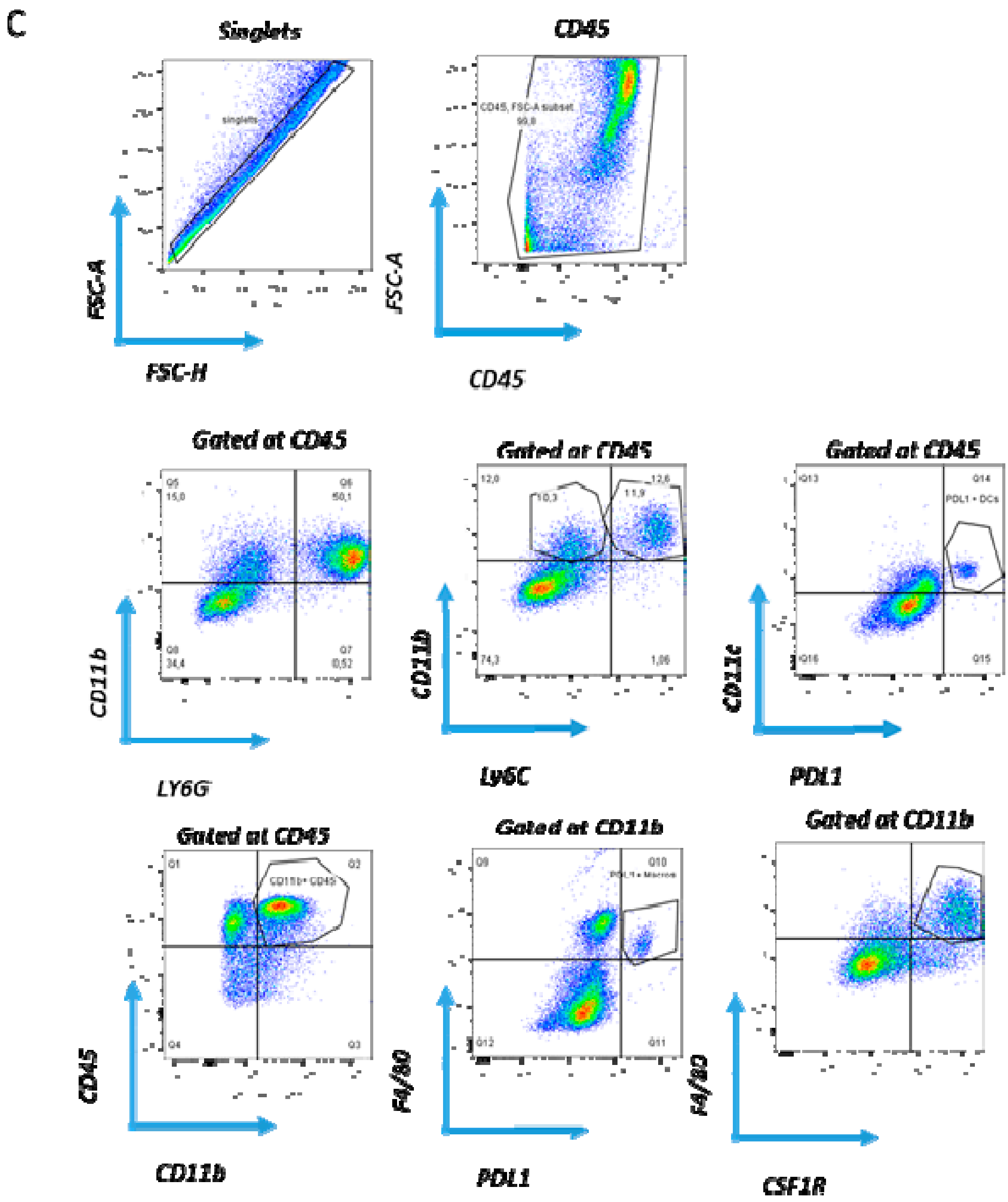

Figure S4. Flow cytometry gating strategy. All cells were first gated on FSC-A/FSC-H in order to select single cells for the subsequent analyses. White blood cells were subsequently selected from the FSC/CD45 plot. (**A**) These cells were further gated for CD11b, or CD11c for monocytes or dentritic cells, respectively or (**A**) CD11b/F4/80, CD11c/MHCII, (C) CD11b/Ly6C and CD11b/Ly6G for MDSCs. Among CD11b/F4/80 gate, cells were further subgated for the subsets of interest, namely (**A**) IL10, IL12 and (**C**) PDL1, CSF1r expressing cells were determined. (**B**) Total lymphocytes were first gated on a CD45/CD3 plot and then gated on the CD4 + (b) or CD8 + population. These were then further gated for the subsets of interest, namely, CD4 + FOXP3 Tregs, CD8+GranzB+, and CD8+PD1+T cells. Data were analyzed using FlowJo software, and population frequencies were expressed as indicated.

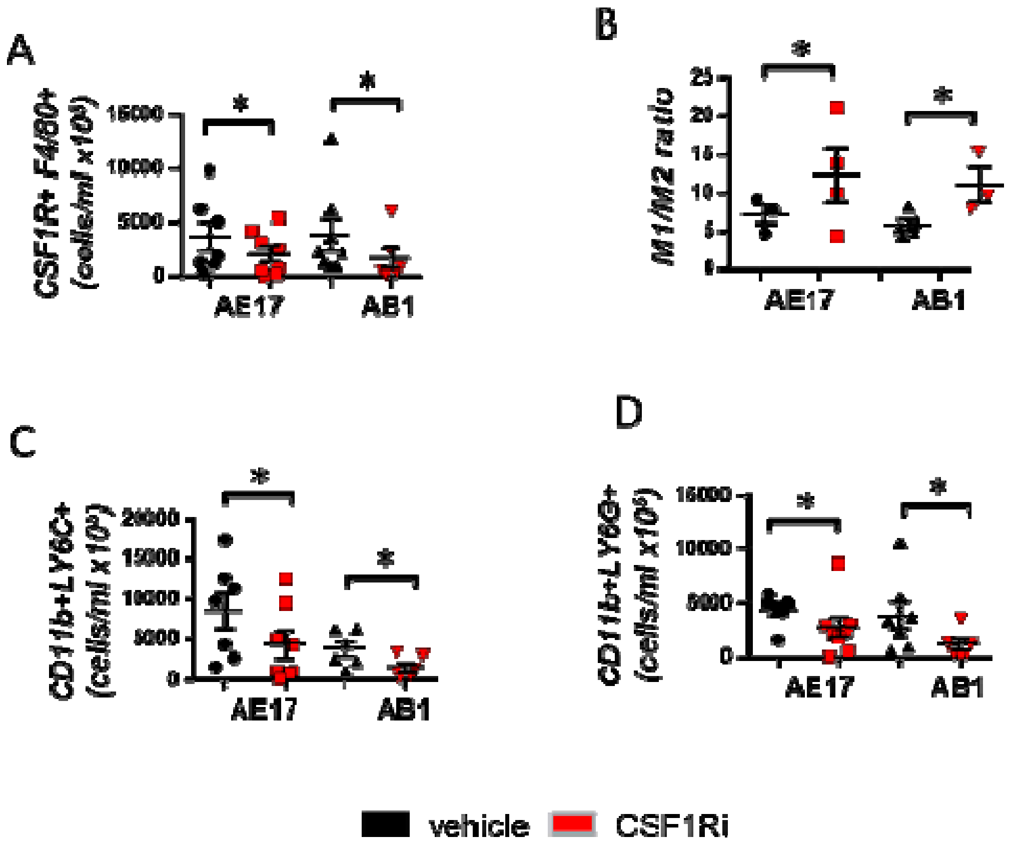
A
CSF1R+ F4/80+ (cells/ml x10⁶)
15000
10000
5000
0
AE17
AB1
B
M1/M2 ratio
25
20
15
10
5
0
AE17
AB1
C
CD11b+LY6C+ (cells/ml x10⁶)
20000
15000
10000
5000
0
AE17
AB1
D
CD11b+LY6G+ (cells/ml x10⁶)
15000
10000
5000
0
AE17
AB1
vehicle
CSF1Ri

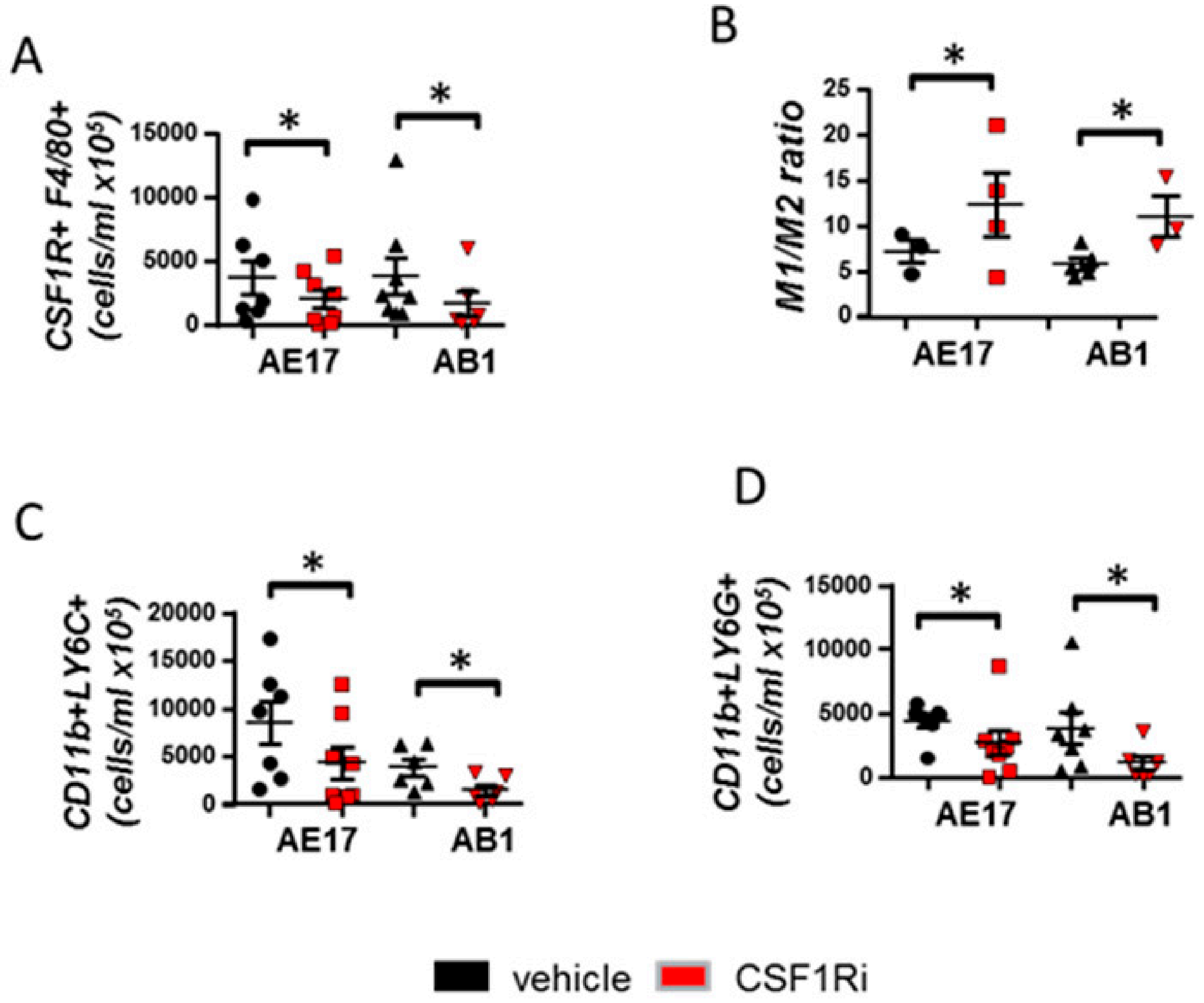

Figure S5. CSF1R inhibition reduces CSF1R+ macrophage numbers in mesothelioma associated pleural effusion and favors M1 TAMs polarization. Pleural fluid cells from vehicle or CSF1Ri treated animals were analyzed for (**A**) CSF1R+ F480+ macrophages by flow cytometry. (**B**) M1/M2 polarization of TAMs was evaluated according to their IL-12/IL-10 expression ratio. Pleural fluid Mo-MDSC (**C**) and PMN-MDSC (**D**) populations were determined. Data are presented as mean ± SEM, n = 5–8, * $p < 0.05$ compared to vehicle.

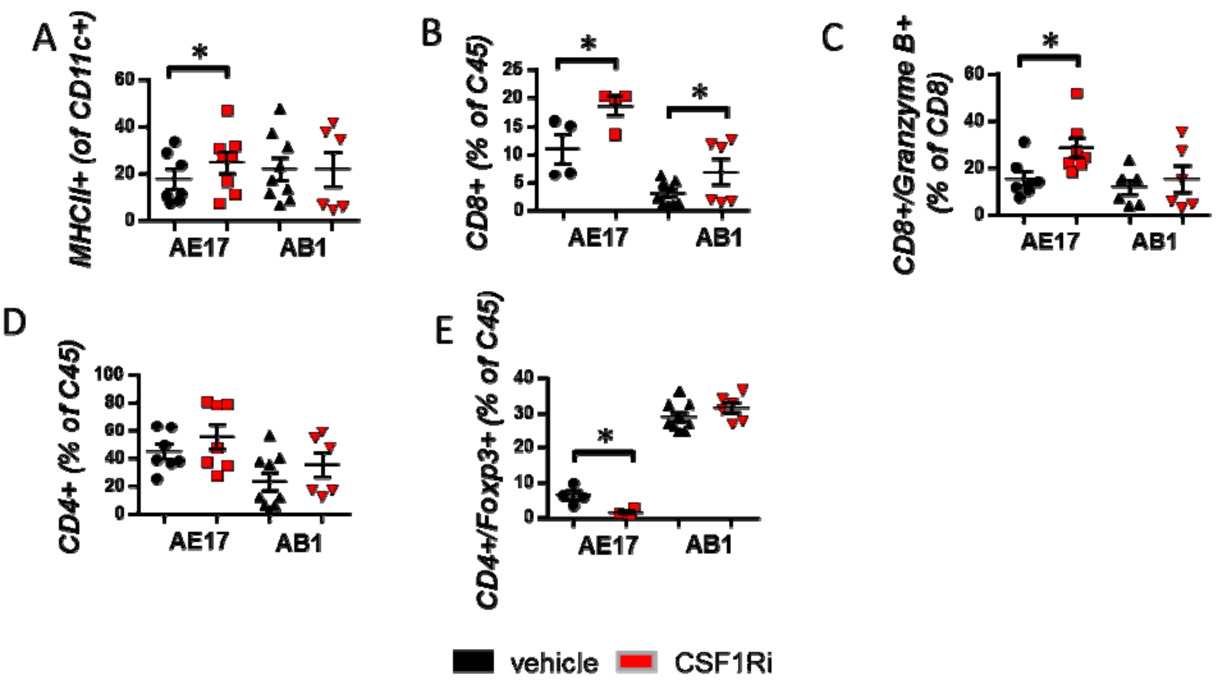

Figure S6. CSF1R inhibition enhances DC activation, favors CD8 lymphocyte recruitment and activation and reduces Treg in mesothelioma associated pleural effusion. Pleural fluid was evaluated as for the presence of activated (MHCII+) dendritic cells (**A**) total (**B**) and activated (Granzyme B+) (**C**) CD8+ cells. Total CD4 (**D**) as well as suppressive T regulatory (Foxp3+) CD4 populations were also quantified. Data are presented as mean ± SEM, $n = 5–8$, * $p < 0.05$ compared to vehicle.

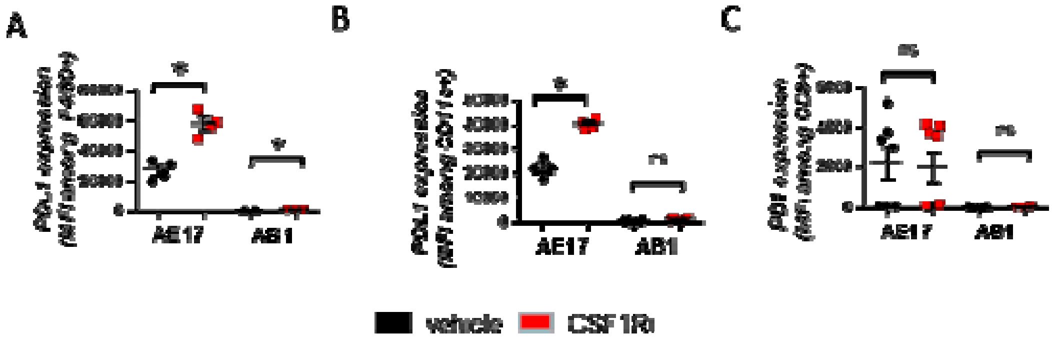

Figure S7. CSF1R blockade up-regulates PDL1 expression by pleural fluid Macrophages and DCs. Mean expression of PD-L1 was determined among pleural fluid F4/80+ macrophages (**A**) and DCs (**B**) using flow cytometry.(**C**) PD-1 expression was evaluated in CD8+ lymphocyte populations of the two groups. Data are presented as mean ± SEM, $n = 5–8$, * $p < 0.05$ compared to vehicle. Data are presented as mean ± SEM, $n = 5–8$, * $p < 0.05$ compared to vehicle.

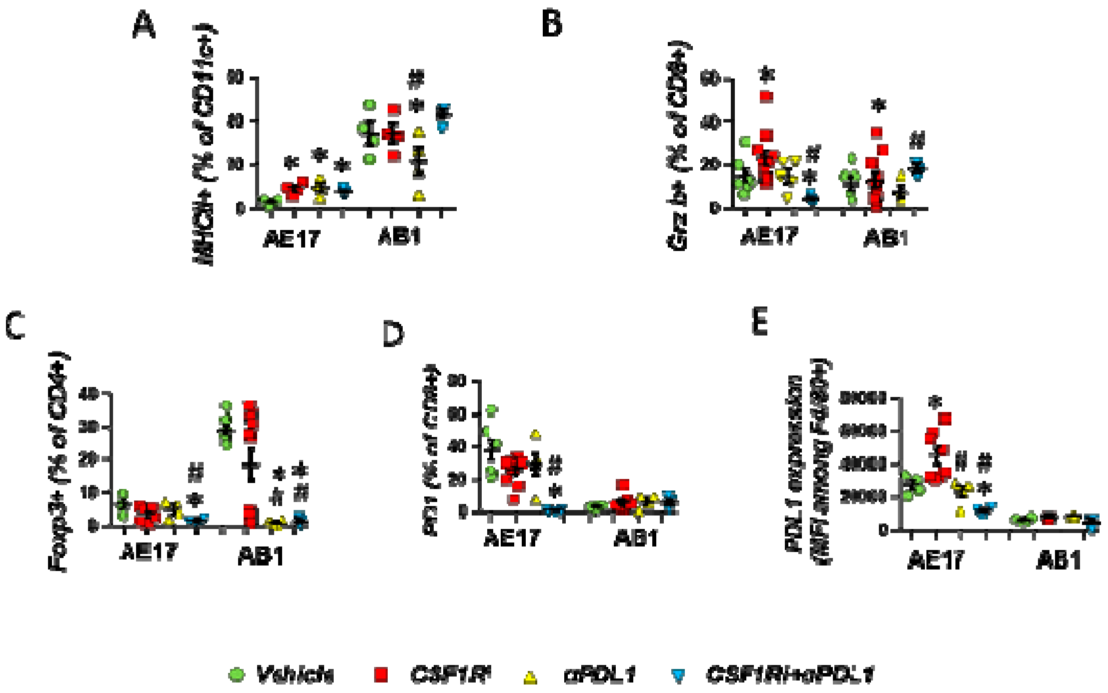

Figure S8. Combined CSF1R and PDL1 blockade significantly enriches pleural fluid with activated CD8 and DC populations and reduces accumulation of suppressive ones. AE17 and AB1 cells were intrapleurally injected into syngeneic C57Bl/6 and Balb/c mice respectively. Mice were administered with CSF1Ri (200 mg/kg b.w, p.o once daily), anti-PDL1 (200 μg/dose i.p) every 3 days, or received both therapies on the respective days. Pleural fluid was evaluated as for the presence of (**A**) activated (MCHII) DCs and (**B**) activated (Granzyme B+) CD8+ lymphocytes. Suppressive (**C**) Tregs (**D**) PD1+/CD8+ lymphocytes and (**E**) PDL1+/F4/80+ macrophage populations were quantified. Data are presented as mean ± SEM, n = 5–8, * $p < 0.05$ compared to CSF1Ri group, # $p < 0.05$ compared to anti-PDL1 group.a.
